# Sexual Dimorphism in the Chemical Composition of Male and Female in the Dioecious Tree, *Juniperus communis* L., Growing under Different Nutritional Conditions

**DOI:** 10.3390/ijms21218094

**Published:** 2020-10-30

**Authors:** Mariola Rabska, Emilia Pers-Kamczyc, Roma Żytkowiak, Dawid Adamczyk, Grzegorz Iszkuło

**Affiliations:** 1 Institute of Dendrology, Polish Academy of Sciences, Parkowa 5, 62-035 Kórnik, Poland; epk@man.poznan.pl (E.P.-K.); romazyt@man.poznan.pl (R.Ż.); dawadamczyk@man.poznan.pl (D.A.); iszkulo@man.poznan.pl (G.I.); 2 Institute of Biological Sciences, University of Zielona Góra, Prof. Z. Szafrana 1, 65-516 Zielona Góra, Poland

**Keywords:** nutrition limitation stress, total nonstructural carbohydrates, starch, phenolic compounds, carbon, nitrogen, C:N ratio, macroelements, dioecious plants, sexual dimorphism, *Juniperus communis* L.

## Abstract

We hypothesized that female and male individuals of the dioecious tree species, *Juniperus communis*, exhibit different strategies of resource allocation when growing under stress conditions. To test this hypothesis, we performed a two-year pot experiment on plants exposed to different levels of nutrient availability. Analysis of the plants revealed a higher concentration of carbohydrates, carbon, and phenolic compounds in needles of female plants, indicating that females allocate more resources to storage and defense than males. This difference was independent of nutrient availability. Differences in carbohydrates levels between the sexes were most often significant in June, during the most intensive phase of vegetative growth in both sexes, but could also be attributed to female resources investment in cone development. A higher level of nitrogen and other macroelements was observed in males than in females, which may have been connected to the accumulation of resources (nitrogen) for pollen grain production in males or greater allocation of these elements to seeds and cones in females. The interaction between sex and soil fertilization for the C:N ratio may also indicate sex-specific patterns of resource allocation and utilization, which is impacted by their availability during specific periods of *J. communis* annual life cycle.

## 1. Introduction

Females of dioecious plants are often reported to show a greater reproductive effort than male plants [1,2] and this effort is more resource-dependent [3]. Females produce resource-consuming seeds and fruits (or cones) that carry substantial cost and for this reason, their reproductive effort can be greater than in males, despite the fact that males often produce a greater number of flowers (e.g., [4]). *Juniperus communis* L., a dioecious tree or shrub common in the northern hemisphere, is an example of a species that exhibits a greater reproductive effort in females than in males [5]. Due to that, females in this species are thought to be more prone to suffer from environmental stress than males. Other studies show that in dioecious species multiple stresses impact female plants more severely than male plants [6]. In this regard, females are more susceptible inter alia to drought [6,7,8], herbivory [6], and in some dioecious species, they are also affected by nutritional limitations [9].

The differences in stress resistance observed between male and female plants can result in greater female mortality [7]. This, however, is attributed also to the greater reproductive effort of females [10,11]. As a consequence, this can lead to a male-biased sex ratio. Lower stress tolerance in females has also been reported to influence the geographical range of populations [12].

The different reproductive roles of male and female individuals not only lead to the generation of different reproductive structures, but they are also connected with the secondary sexual dimorphism, i.e., not directly connected with generative organs. It can be observed inter alia as different resource demands. For example, male reproductive structures are reported to require more nitrogen for pollen production while females require more carbon for seed development [13]. In this regard, greater concentrations of carbohydrates have been reported in females by many authors (e.g., [14,15]). Moreover, different concentrations of secondary metabolites have also been reported in some dioecious woody plants [16].

In addition to inherent differences in resource composition in males and females, resource allocation can vary between both sexes as the response of females to greater reproductive costs [17], but also as the response and adaptation to local environmental conditions [18]. In a longer time perspective, resource allocation to reproduction in females can be greater even if males flower earlier and more frequently than females [17]. As a consequence, males can use a greater amount of resources available for vegetative growth than females [17]. In regards to *J. communis*, however, it is important to note that female cones are able to photosynthesize as it was observed in *Taxus baccata* L. green arils [19] and they compensate at least a part of their production costs [20].

Stress conditions (e.g., drought, nutrient deficiency) can affect allocation strategies differently in males and females [21]. The depletion of nutrients stored in stems can have a more severe effect on reproduction in females than in males [9]. In fact, differences in the sex-related allocation of resources in dioecious species growing under different nutritional conditions have been reported [14,22]. Despite some research concerning differences between sexes, further studies are needed pertaining to life-history traits and resource allocation strategies [23].

Most of the nutrients obtained by plants originate in the soil and soil fertilization influences the overall physiology of a plant. Nitrogen and phosphorus are the most limiting nutrients in plants [24]. Nitrogen deficiency has been demonstrated to affect the expression of genes connected with N and carbohydrate metabolism, secondary metabolism, and stress response [25]. Moreover, higher nitrogen availability increases the uptake of phosphorus [26].

Seed and fruit (or female cone) formation requires a significant amount of macro- and microelements. One of the most abundant elements in seeds are P and Ca, while K is present to a lesser extent [27]. Seed and accompanied structure formation requires also additional resources. Their reservoirs in plants are mainly starch and soluble sugars. Starch synthesis serves as a method of storing energy in plants, while soluble sugars are typically used for ongoing metabolism [28]. A higher concentration of soluble sugars can be achieved by starch hydrolysis, while this can be induced in response to the energy needs for other physiological processes [29]. Moreover, stress can result in lower starch levels [30].

In conclusion, dioecious plants often show secondary sexual dimorphism and the sexes differ in reproductive effort, resource demands, and allocation as well as in their response to stress. We feel that studies about the influence of long-term nutrition limitation on resource storage strategies in both sexes are needed, especially regarding particular phenological phases. Previous studies showed that *Juniperus communis* is a good model species for the study of sexual dimorphism in dioecious plants. Significant differences in growth rate, morphology, and ecophysiology were found in this species [5,31,32], but no detailed analysis of chemical composition, considering both differences between sexes and soil fertilization treatment, have been conducted. Moreover, similarly to many other dioecious species, a decline in population size of *J. communis* and a sex ratio bias are observed [33,34,35,36] which makes such studies even more necessary.

In the current study, we attempted to determine if male and female plants of *J. communis* differ in their response to long-term nutrient limitations in regards to the synthesis and allocation of carbohydrates, macroelements, and phenolic compounds in leaves. At the same time, we focused on this response during the annual cycle of growth. We hypothesized that:

(1) Females allocate more resources to storage and defense than males. Therefore, female leaves have a greater concentration of carbon, carbohydrates, and C:N ratio, as well as phenolic compounds, but a lower concentration of other macroelements.
(2) Differences between sexes are most evident when plants are grown under nutrient limited conditions.
(3) Differences between male and female plants will be most pronounced after strobili opening when females produce seeds and accompanying structures and during the most intensive period of vegetative growth. Differences would also be evident in winter (December) when additional stresses, such as low temperatures, occur.

## 2. Results

### 2.1. Effect of Soil Fertilization

No differences in the concentration of soluble sugars were observed between fertilized (F) and nonfertilized (NF) plants. Starch and total nonstructural carbohydrates (TNC) concentrations, however, were higher in NF plants in March during both years of the study (2014 starch: NF 5.06 ± 0.61, F 1.75 ± 0.19, *p* < 0.0001; 2014 TNC: NF 13.36 ± 0.70, F 10.23 ± 0.21, *p* = 0.0002; 2015 starch: NF 5.35 ± 0.57, F 2.61 ± 0.38, *p* = 0.0011; 2015 TNC: NF 12.66 ± 1.08, F 8.99 ± 0.69, *p* = 0.0099) and in June 2014 (starch: NF 7.66 ± 0.97, F 5.32 ± 0.67, *p* = 0.0234; TNC: NF 13.13 ± 0.99, F 10.76 ± 0.72, *p* = 0.0156). The concentration of total phenolic compounds was higher in NF plants in March 2015 (NF 100.73 ± 4.14, F 80.44 ± 5.78, *p* = 0.0074) and December 2015 (NF 198.85 ± 5.65, F 177.98 ± 5.39, *p* = 0.0061) (Tables S1 and S2).

Fertilized plants had a higher concentration of C in March of both years (2014 NF 47.39 ± 0.12, F 47.94 ± 0.09, *p* = 0.0019; 2015 NF 47.65 ± 0.15, F 48.44 ± 0.16, *p* = 0.0008) and in September 2014 (NF 48.43 ± 0.15, F 48.85 ± 0.16, *p* = 0.0309). They also had a higher concentration of C in June 2015 (NF 47.52 ± 0.09, F 47.83 ± 0.07, *p* = 0.013). N concentration and the C:N ratio were both significantly different in plants growing under different nutritional conditions over the entire course of the study. Notably, the concentration of N was higher in F plants, and the C:N ratio was higher in NF plants.

Fertilized plants had a significantly higher concentration of P than NF plants during four sampling times (March 2014 NF 0.18 ± 0.01, F 0.24 ± 0.01, *p* = 0.0019; July 2014 NF 0.14 ± 0.01, F 0.16 ± 0.01, *p* = 0.0134; December 2014 NF 0.18 ± 0.01, F 0.25 ± 0.01, *p* < 0.0001; March 2015 NF 0.16 ± 0.01, F 0.26 ± 0.01, *p* < 0.0001), while the results were reversed in September 2015 (NF 0.22 ± 0.01, F 0.21 ± 0.01, *p* = 0.0462). Differences in the concentration of K varied, with F plants having a higher concentration in September 2014 (NF 0.80 ± 0.02, F 0.90 ± 0.04, *p* = 0.0020) but a lower concentration in December 2014 (NF 0.67 ± 0.02, F 0.57 ± 0.02, *p* = 0.0012), followed by a higher concentration in F plants again in March 2015 (NF 0.64 ± 0.02, F 0.78 ± 0.02, *p* = 0.0002), and then a higher concentration in NF plants in July 2015 (NF 0.76 ± 0.02, F 0.68 ± 0.02, *p* = 0.0098). The concentration of Ca was higher in F plants over the course of the entire study, however, the concentration of Mg was significantly higher in F plants only in March 2015 (NF 0.18 ± 0.01, F 0.20 ± 0.01, *p* = 0.0255) (Table S2).

### 2.2. Effects of Sex and Sex × Soil Fertilization Interactions

**Figure 1:**
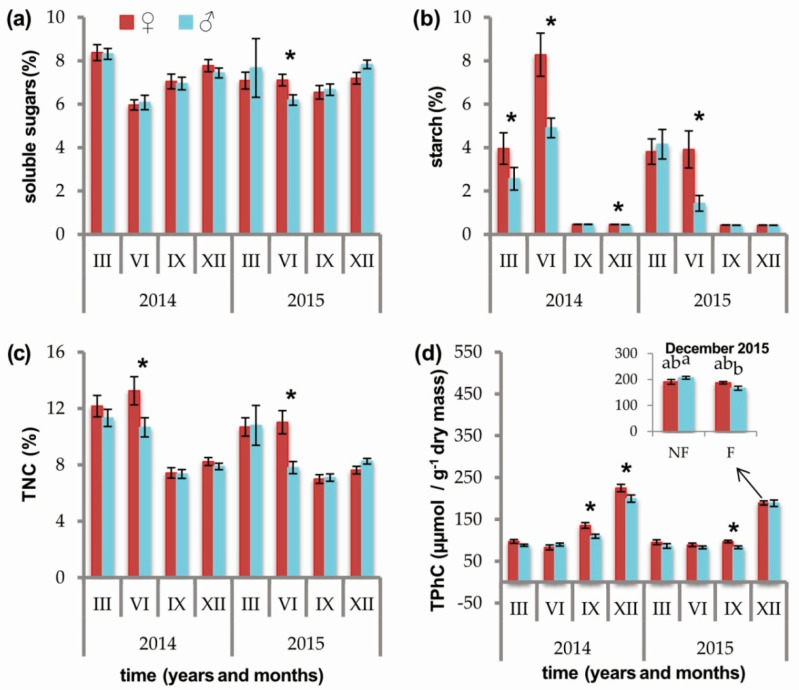
Effect of sex (female (red) vs. male (blue)) on the concentration of (**a**) soluble sugars (%); (**b**) starch (%); (**c**) total nonstructural carbohydrates (TNC; %); (**d**) total phenolic compounds concentration (TPhC; µmol/ g^−1^ dry mass) in needles of *Juniperus communis* measured at eight time points over the course of two years. Asterisks indicate significant influence of sex (*p* < 0.05). Values for time points with a significant interaction between sex and soil fertilization treatment (fertilized F vs. nonfertilized NF) are illustrated in the inset.

Female individuals exhibited a higher concentration of soluble sugars, starch, TNC, and total phenolic compounds (TPhC) than male individuals (Figure 1, Table S1). The concentration of soluble sugars in females, however, was only higher in July 2015 (♀ 7.11 ± 0.27, ♂ 6.19 ± 0.24, *p* = 0.0197) (Figure 1a, Table S2). At this sampling time point (July 2015), the concentration of starch and TNC was also higher in female plants than in male plants (starch: ♀ 3.91 ± 0.85, ♂ 1.43 ± 0.36, *p* = 0.0103; TNC: ♀ 11.02 ± 0.82, ♂ 7.80 ± 0.43, *p* = 0.0033). Those differences were also evident in July 2014 (starch: ♀ 8.28 ± 0.99, ♂ 4.91 ± 0.45, *p* = 0.0031; TNC: ♀ 13.25 ± 1.00, ♂ 10.66 ± 0.68, *p* = 0.0107) (Figure 1b,c). The concentration of starch was also higher in females than in males in March 2014 (♀ 3.95 ± 0.73, ♂ 2.56 ± 0.52, *p* = 0.0072) and December 2014 (♀ 0.46 ± 0.00, ♂ 0.44 ± 0.00, *p* = 0.0465) (Figure 1b). The concentration of TPhC was higher in females in September in both 2014 (♀ 135.44 ± 6.76, ♂ 109.66 ± 4.84, *p* = 0.0046) and 2015 (♀ 97.02 ± 3.07, ♂ 83.12 ± 3.36; *p* = 0.0066), as well as December 2014 (♀ 224.85 ± 8.81, ♂ 199.57 ± 8.59, *p* = 0.0350) (Figure 1d). Significant variability in the interaction between sex and soil fertilization treatment was observed for TPhC in December 2015. The highest concentration of TPhC occurred in NF males (NF♂ 206.81 ± 5.55(a)) and was significantly lower in F males (F♂ 166.47 ± 7.88 (b)). The values in both of these groups, however, were not significantly different than groups of females (NF♀ 190.90 ± 9.19 (ab), F♀ 187.58 ± 4.96 (ab), *p* = 0.0170) (Figure 1d, Tables S1 and S2).

**Figure 2:**
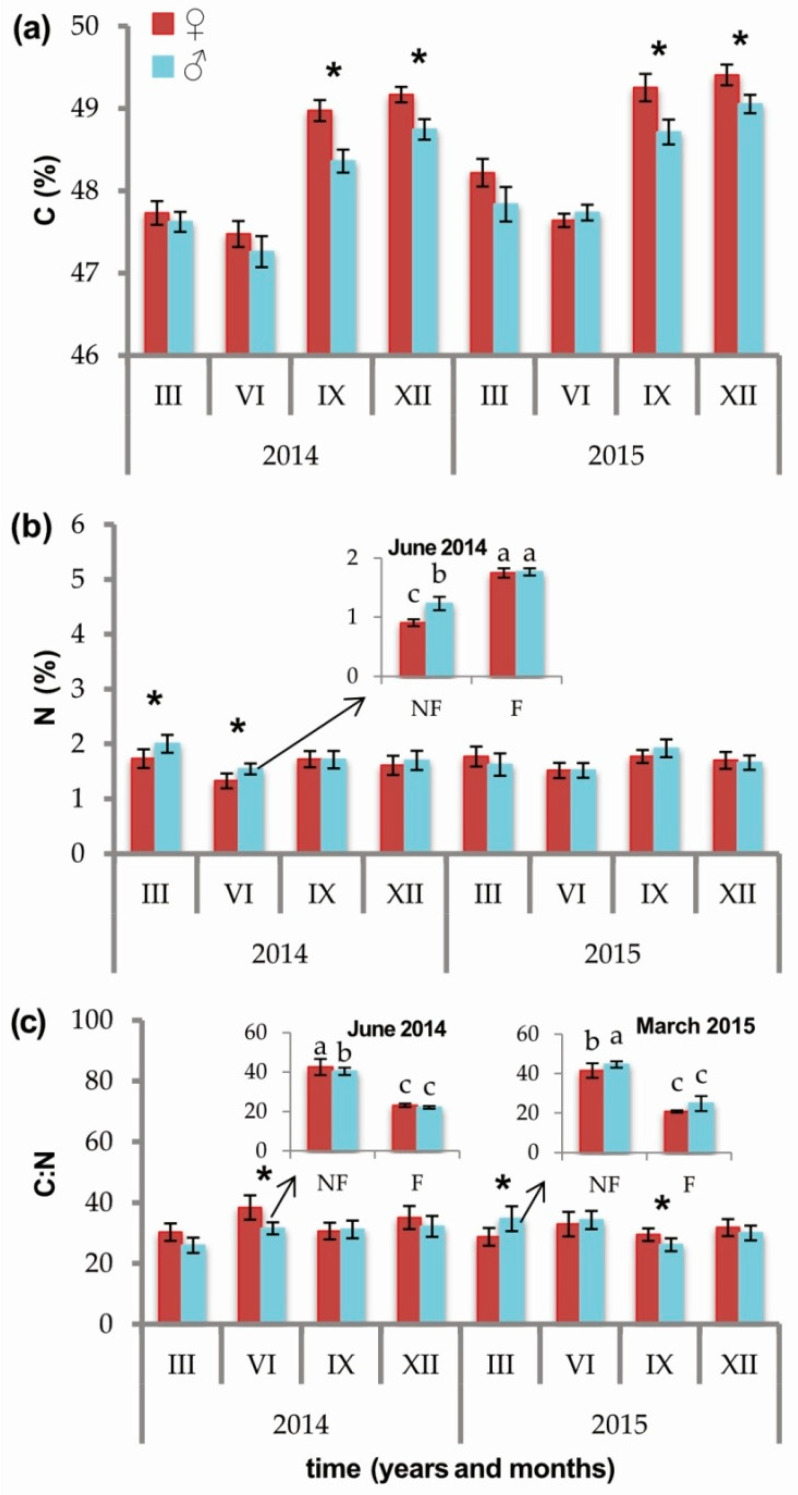
Effect of sex (female (red) vs. male (blue)) on the concentration of (**a**) carbon (%); (**b**) nitrogen (%); (**c**) C:N ratio in needles of *Juniperus communis* measured at eight time points over the course of two years. Asterisks indicate significant influence of sex (*p* < 0.05). Values for time points with a significant interaction between sex and soil fertilization treatment (fertilized F vs. nonfertilized NF) are illustrated in the insets.

Female individuals exhibited a higher concentration of C than males (Figure 2a) in the colder months, i.e., in September 2014 (♀ 48.97 ± 0.13, ♂ 48.36± 0.14, *p* = 0.0027) and 2015 (♀ 49.25 ± 0.17, ♂ 48.71 ± 0.15, *p* = 0.0251), as well as in December 2014 (♀ 49.17 ± 0.09, ♂ 48.75 ± 0.12, *p* = 0.0112) and 2015 (♀ 49.41 ± 0.13, ♂ 49.05 ± 0.11, *p* = 0.0407) (Figure 2a). In contrast, males had a higher concentration of N in March 2014 (♀ 1.73± 0.17, ♂ 2.00 ± 0.16, *p* = 0.0560) and June 2014 (♀ 1.32 ± 0.14, ♂ 1.54 ± 0.10, *p* = 0.0140) (Figure 2b). Notably, a significant interaction between sex and soil fertilization treatment was observed for N concentration in June 2014, where F plants of both sexes had the highest N concentration (F♀ 1.75 ± 0.08(a) and F♂ 1.77 ± 0.06(a)), and whose values were significantly different from NF plants. The lowest concentration of N, however, was observed in NF females (NF♀ 0.91 ± 0.06(c) and NF♂ 1.23 ± 0.11(b), *p* = 0.0242) (Figure 2b, Tables S1 and S2).

The C:N ratio was higher in males in March 2015 (♀ 28.73 ± 2.91, ♂ 34.70 ± 4.09, *p* = 0.0152), and higher in females in June 2014 (♀ 38.38 ± 4.00, ♂ 31.52 ± 1.98, *p* = 0.0016) and September 2015 (♀ 29.44 ± 2.08, ♂ 26.12 ± 2.11, *p* = 0.0234) (Figure 2c). The C:N ratio exhibited significant differences in regards to the interaction between sexes and soil fertilization treatment in June 2014 and in March 2015. Both sexes had the lowest C:N ratio in F plants in June 2014 (F♀ 27.13 ± 1.45(c), F♂ 27.12 ± 0.95(c)), when NF females had the highest C:N ratio (NF♀ 53.56 ± 3.32(a)), a value that was significantly greater than NF males (NF♂ 39.85 ± 3.76(b), *p* = 0.0027) (Tables S1 and S2). Fertilized plants still had the lowest C:N ratio regardless of sex in March 2015, however, the highest values were observed in NF males and intermediate values in NF females (C:N ratio: NF♀ 41.53 ± 3.69(b), F♀ 20.78 ± 0.58(c), NF♂ 44.62 ± 1.61(a), F♂ 24.80 ± 3.80(c), *p* = 0.0207) (Figure 2c, Tables S1 and S2). 

**Figure 3:**
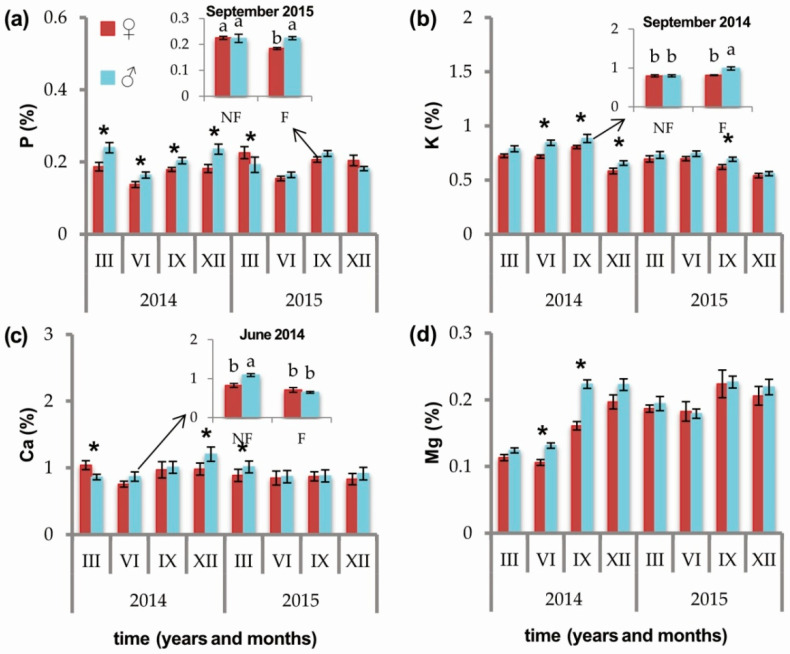
Effect of sex (female (red) vs. male (blue)) on the concentration of (**a**) phosphorus (%); (**b**) potassium (%); (**c**) calcium (%); (**d**) magnesium (%) in needles of *Juniperus communis* measured at eight time points over two years. Asterisks indicate significant influence of sex (*p* < 0.05). Values for time points with a significant interaction between sex and soil fertilization treatment (fertilized, F vs. nonfertilized, NF) are illustrated in the insets.

Phosphorus concentration was higher in male than in female individuals during the first four sampled time points (March 2014 ♀ 0.19 ± 0.01, ♂ 0.24 ± 0.01, *p* = 0.0041; July 2014 ♀ 0.14 ± 0.01, ♂ 0.16 ± 0.01, *p* = 0.0186; September 2014 ♀ 0.18 ± 0.01, ♂ 0.20 ± 0.01, *p* = 0.0255; December 2014 ♀ 0.18 ± 0.01, ♂ 0.24 ± 0.01, *p* = 0.0003) and was then reversed (higher in females) at the fifth time point (March 2015 ♀ 0.23 ± 0.02, ♂ 0.19 ± 0.02, *p* = 0.0483) (Figure 3a). A significant interaction between sex and soil fertilization treatment was observed for P levels in September 2015. At that time, fertilized females had a significantly lower concentration of P than any of the other sample groups (phosphorus: NF♀ 0.23 ± 0.01 (a), F♀ 0.18 ± 0.00 (b), NF♂ 0.22 ± 0.02 (a), F♂ 0.22 ± 0.01 (a), *p* = 0.0330) (Figure 3a, Tables S1 and S2).

The concentration of K was higher in male individuals during periods corresponding to the accumulation of biomass in roots, during both years (September 2014 ♀ 0.80 ± 0.01, ♂ 0.88 ± 0.04, *p* = 0.0080; September 2015 ♀ 0.62 ± 0.02, ♂ 0.69 ± 0.02, *p* = 0.0238) (Table S2). K levels were also higher in males in July 2014 (♀ 0.72 ± 0.02, ♂ 0.84 ± 0.02, *p* = 0.0002) and December 2014 (♀ 0.58 ± 0.03, ♂ 0.66 ± 0.02, *p* = 0.0126) (Figure 3b). A significant interaction between sex and soil fertilization treatment was observed in September 2014, when fertilized males had a significantly higher concentration of K than any of the other sample groups (potassium: NF♀ 0.80 ± 0.03 (b), F♀ 0.81 ± 0.01 (b), NF♂ 0.80 ± 0.03 (b), F♂ 0.99 ± 0.04 (a), *p* = 0.0091) (Figure 3b, Tables S1 and S2).

The concentration of calcium was higher in female individuals prior to the period of strobili opening in March 2014 (♀ 1.04 ± 0.07, ♂ 0.86 ± 0.04, *p* = 0.0001) and then reversed with higher levels of calcium in males prior to strobili opening in March 2015 (♀ 0.89 ± 0.09, ♂ 1.01 ± 0.09, *p* = 0.0455) and during the dormant period (December 2014 ♀ 0.98 ± 0.09, ♂ 1.20 ± 0.11, *p* = 0.0231) (Figure 3c). A significant interaction between soil fertilization treatment and sex in the level of Ca was observed in July 2014, where NF males had a significantly higher level of Ca than any of the other sample groups (calcium: NF♀ 0.83 ± 0.05 (b), F♀ 0.71 ± 0.06 (b), NF♂ 1.09 ± 0.04 (a), F♂ 0.65 ± 0.02 (b), *p* = 0.0043) (Figure 3c, Tables S1 and S2).

The concentration of magnesium was higher in males in July 2014 (♀ 0.11 ± 0.00, ♂ 0.13 ± 0.00, *p* = 0.0004) and September 2014 (♀ 0.16 ± 0.01, ♂ 0.22 ± 0.01, *p* < 0.0001) (Figure 3d, Table S2).

## 3. Discussion

The different functional roles of males and females in dioecious species may result in different strategies of resource allocation (e.g., [37,38]). Results of our present study confirmed that females allocate more resources to storage and defense than males, thus males exhibited a lower accumulation of starch, TNC, carbon, and TPhC than females in their needles (leaves). Those concentrations of carbohydrates and C, however, were not related to availability of nutrients as initially hypothesized. Our results on carbohydrates did confirm, as we hypothesized, that differences between both sexes generally appear during the time directly after strobili opening (in June) when the most intensive period of vegetative growth occurs in both sexes of plants, but females are also allocating resources into cone development. Seed production requires significant resource demands which can result in increasing stress levels in plants [39], and the increase of accumulation of nonstructural carbohydrates is directly associated with plant response to stress conditions [40]. In our study, however, no significant interaction between sex and soil fertilization treatment was observed for carbohydrate accumulation, so the differences in carbohydrate levels reflect an allocation strategy rather than a stress response.

### 3.1. Carbohydrates

In our study, low nutrient availability resulted in increased accumulation of TNC and starch in needles of both sexes. These findings are similar to the results obtained in *Salix paraplesia* where low nutrient availability resulted in an increase in the accumulation of nonstructural carbohydrates and, similar to our findings, the concentration was higher in females and no interaction between sex and nutrient availability was observed for carbohydrate levels [38]. Lack of interaction between sex and soil fertilization in our studies result in rejection of our second hypothesis. Different studies show that female plants had higher levels of soluble sugars than male plants in leaves of *Populus cathayana* when they were grown under elevated CO_2_ and N deposition [14]. Similarly, female plants of *Tinospora cordifolia* had higher concentrations of total sugars and starch than male plants in different seasons [15]. Additional comparative studies with a greater number of species need to be conducted to definitely determine if a greater accumulation of carbohydrates in females of dioecious perennial plants is a general rule. Studies in species that exhibit sex plasticity, however, have demonstrated that plants which produce and maintain female generative organs have higher long-term concentrations of TNC [41]. We assume that the greater accumulation of carbohydrates in female plants of *J. communis* is related to the different reproductive roles of male and female plants rather than to a stress response. This conclusion is supported by a similar carbohydrate accumulation response in both sexes when they were grown under different levels of soil fertilization, as well as the lack of a statistically significant interaction between sex and soil fertilization treatment.

The accumulation of soluble sugars is part of a short-time response to stress conditions [28]. Nutrient availability did not impact the concentrations of soluble sugars, indicating that their synthesis does not play a role in the response of *J. communis* to different levels of soil fertilization, but rather that differences in soluble sugars observed between male and female individuals in July 2015 are connected to the different energy demands required by female plants for cone development. This observation stands in contrast to a study on *Populus cathayana*, where greater N deposition in the environment resulted in a greater shift from starch to soluble sugars in females than in males, while excessive accumulation of starch and soluble sugars was observed in females under control conditions [42].

Male individuals can adopt an energy-saving strategy when they grow in nutrient deficient conditions [43]. In our current study, however, a significant interaction between soil fertilization treatment and sex in the concentration of carbohydrates was not observed, contrary to our second hypothesis. Instead, our results indicated that females had a higher accumulation of carbohydrates than male plants; independent of nutrient availability.

Starch represents an energy reserve, therefore, its concentration is generally low during active periods of plant growth and high during the winter when plants are dormant [15,44]. In contrast, the concentration of total soluble sugars is generally the highest in summer [15], but not always [45]. In our study, the lowest concentrations of starch were observed in September and December in both years. This phenomenon is observed in coniferous, evergreen species, which in contrast to deciduous trees, maintain their metabolism during winter and in colder months decrease in starch [46,47] and increase in fat was observed [47]. In addition, differences in starch levels were evident between male and female plants in June. Notably, the highest air temperatures also occurred at this time, which may have affected starch accumulation as elevated temperature (more than 25 °C) can inhibit starch synthesis [48]. Additionally, our previous study shows that the high temperatures can also have a differential impact on leaf mass area (LMA) values in male and female plants; with females exhibiting a significantly higher LMA during the hottest month of the two years of the study (females 156.78 ± 3.13 and males 147.35 ± 3.23, *p* = 0.0465) [32]. The thicker lamina present in leaves of female plants, relative to male plants, can better protect cells against a sharp rise in temperature [49]. As a consequence of this protective feature, starch synthesis can be maintained in female plants.

In studies conducted during the same time on male and female plants of *J. communis* it was found that the two sexes accumulate a similar amount of total biomass regardless of nutrient availability, however, both sexes differed in their allocation to aboveground vs. root tissues [50], data not published]. Therefore, it is plausible that the different strategies of carbohydrate allocation (greater TNC, starch, and soluble sugars in females than in males) do not influence plant growth and are probably associated to a greater extent with differences in their allocation to generative functions and defense.

### 3.2. Carbon, Nitrogen, C:N Ratio, and Other Elements

Increased nitrogen deposition has been previously observed to induce an increase in the level of carbon in female individuals of *Populus cathayana*, where the concentration of C was lower in control females than those which received nitrogen supplementation [42]. In that study, however, carbon concentration was independent of nitrogen deposition in males [42]. In our current study, C concentration was higher in females than males, independent of fertilizer treatment, although carbon levels were higher in F plants than in NF plants. These data indicate that despite an increase in C accumulation in fertilized plants, females have mechanisms that allow them to maintain a higher concentration of C, relative to males, even in a nutrient deficient environment.

A higher N concentration was observed in needles of *J. communis* male plants in March and June 2014 and a higher C:N ratio was observed in March 2015. These data can suggest that males require more N than females in order to produce pollen [51], a requirement that could have a significant impact when plants are grown in a low nutrient environment (see Figure 2b,c). As an extension, it is plausible to suggest that nonfertilized males allocate their limited N supply to strobili rather than leaves. Differences in the C:N ratio can be the result of differences in the allocation of these elements between the sexes. Under conditions of elevated CO_2_ and N deposition, a higher C:N ratio in *Populus cathayana* was observed in roots of male individuals whereas the concentration of N in leaves was also greater in males than in females [14]. Consistent with our second hypothesis, a greater C:N ratio was observed in nonfertilized female individuals, relative to nonfertilized males, in June 2014. These data indicate that females are more depleted of nitrogen than males under nutrient-limited conditions, where the nitrogen is probably allocated to seed production. On the other hand, the higher C:N ratio observed in females was not always influenced by nutrient availability, thus providing additional evidence for sex differences in nutrient allocation strategies.

Males require nitrogen to produce pollen and male inflorescences have a greater concentration of nitrogen than female inflorescences [4]. Notably, a lower level of nitrogen was detected in females of *Mercurialis perennis* during the reproductive stage, which was attributed by the authors to nitrogen allocation to seeds [52]. Similar conclusions have also been formulated by Bañuelos and Obeso [8]. Despite the previous demonstration of a greater allocation of N to male cones [13], females allocate more N and P to reproduction than males where those elements are mainly used in seed and fruit production [4]. In accordance to our first hypothesis, this strategy is most likely present in *J. communis* where females exhibit lower concentrations than males in N, as well as the other measured macroelements. Moreover, the concentration of K in female generative structures has been reported to increase during the transition from flowers to seeds and accompanying structures [4]. Notably, differences between male and female plants in the concentration of K in our present study appeared after strobili opening (a process analogous to flowering). This confirms our third hypothesis and indicates that K may be allocated more intensively to generative organs in females during seed formation. Differences in K allocation were also confirmed by results obtained in F and NF plants where fluctuations in the differences between the soil fertilization treatments were observed, indicating that fertilized plants allocate or use K differently, for example, to increase growth.

Low concentrations of nitrogen can influence growth in female plants due to lower photosynthetic capacity [23]. In the current study, the lower levels of nitrogen detected in needles of female plants of *J. communis* may be connected to the lower photosynthetic efficiency of females detected in a previous study [32]. It has been previously suggested that male individuals of *J. thurifera* use available nutrients to increase gas exchange capacity, while females exhibit a long-term strategy and increase N storage, saving N-reserves for reproduction [53]. In this regard, female plants of *Populus cathayana* have been reported to exhibit a higher level of plasticity in N allocation than male plants in response to increased N deposition and use N more efficiently in response to N enrichment [42].

Fertilized females probably use any additional P and K to increase reproduction while fertilized males store excess elements in leaves. Nonfertilized females have no redundant resources and thus have similar concentrations of macroelements as nonfertilized males. This suggestion, however, is contrary to our second hypothesis; as well as the findings of Xia et al. [22] where female plants growing under high P supply exhibited a greater concentration of P in leaves than male plants.

### 3.3. Phenolic Compounds

Female and male individuals can differ in their response to biotic and abiotic stresses. One type of stress response is to increase the concentration of total phenolic compounds (TPhC). In dioecious species, female plants generally accumulate a higher level of TPhC than male plants [3,15,54]. The increased accumulation occurs regardless of habitat [55] and in different seasons [15], however, this is not always the case [52]. Our results are similar to the majority of studies and are in agreement with our first hypothesis. Female plants exhibited a higher concentration of TPhC, and in accordance to our third hypothesis, differences between sexes were the most pronounced in autumn and winter.

Female and male plants can respond differently to stress conditions in regard to TPhC. Drought stress can decrease TPhC in females but not in males [6]. Warmer temperatures can decrease TPhC levels while UV-B radiation can increase the concentration of phenolic glycosides in leaves [54]. Increased levels of UV-B also induce an increase in some phenolic compounds in female plants of *Populus tremula*, but not in males [54]. Moreover, increased temperature and UV increased the concentration of secondary metabolites in female plants of *Salix myrsinifolia* [16].

Warm temperatures and elevated CO_2_ can sometimes induce similar increases in growth and the accumulation of phenolic compounds in both male and female plants [56]. In our present study, however, during the period of the highest temperatures TPhC concentrations were relatively low and did not differ between sexes. On the other hand, fruiting has been demonstrated to reduce the concentration of secondary metabolites [3]. However, it is important to note that this was not observed in our study, where female plants of *J. communis* were found to have a higher level of TPhC than male plants, which do not bear cones.

In our study, NF plants exhibited a higher concentration of TPhC than F plants, indicating that TPhC in *J. communis* is produced as a response to stress associated with nutrient limitation. On the other hand, carbon concentration is higher in F plants while carbohydrates and TPhC concentrations are higher in NF plants, which may indicate that F plants invest carbon differently than stressed plants and probably invest more in different compounds (other than starch and phenolics) than NF plants or have different allocation strategy. It was shown previously that phosphorus availability influence allocation of resources and P-limited plants invested more in constitutive and induced chemical defenses while fertilized plants invested more in growth [57].

A recent study of Song et al. [25] reported that female individuals of *Populus cathayana* exhibited a higher level of secondary metabolite activity than males in response to nitrogen deficiency. Males plants, however, had greater stress tolerance, and did not invest as much in defense as females [25]. A similar response was present in our study where male plants had a lower concentration of TPhC than female plants during the winter months when cold stress was severe.

We found that male and female plants can sometimes (December) respond differently to nutrient limitation and that a higher concentration of TPhC was present in NF males compared to F males. However, no differences were apparent between females growing under different soil fertilization treatments. These finding are in contrast to the study of Randriamanana et al. [58] where no differences between male and female plants of *Populus tremula* were found in regards to the concentration of phenolics in plants grown in different levels of N and P fertilization. Our findings indicate that in *J. communis*, higher levels of phenolic compounds in female plants can be independent of nutrient conditions, while in males, differences can be connected to the nutrient stress response. We assume that the main function of phenolics in *J. communis* is to protect plant from the adverse impact of low temperatures because of the observed elevation of TPhC levels in autumn and winter [59].

## 4. Materials and Methods

### 4.1. Plant Material

Fifty shoots of *Juniperus communis* L. were collected in 2012 from each of 10 male and 10 female mature plants growing in the Rokita forest district, Western Pomerania, Poland. Shoots were taken from the middle part of branches and were chosen from branches that had no evidence of reproductive development. A total of 1000 shoots were rooted, transferred to the Institute of Dendrology, Polish Academy of Sciences in Kórnik, Poland and maintained in a greenhouse in 10-litre pots under two-meter-high scaffolding covered with a shading net. The shading net reduced full sunlight by 50% and it was confirmed by the measurements of the relative photosynthetic photon flux density inside the shaded canopy. For this purpose a line quantum sensor (Apogee Inc., Logan, UT, USA) was used according to the method of Messier and Puttonen [60]. The soil substrate used in the experiment was taken from a mixed broadleaved forest. A total of 10% of the soil volume originated from the location in which the maternal plants grew to provide the introduction of mycorrhizae. Pots were watered separately by an automatic irrigation system. Plants from each group of soil fertilization treatment were irrigated with different amounts of water as soil fertilization increases the growth of the plant and water demands. To provide plants with sufficient amount of water and avoid water stress fertilized individuals got twice the volume of water as nonfertilized plants. Plants were watered each day to keep the medium soil moisture during the whole vegetation season.

### 4.2. Experimental Design and Sampling

Potted seedlings of both sexes were randomly divided into two groups in March 2013 with the same number of male (♂) and female (♀) plants in each of the two groups. One group was provided fertilizer (F) while the other group was nonfertilized (NF). Plants in the F group were fertilized every year in May, after strobili opening had occurred. The fertilizer application consisted of five grams of slow-release fertilizer (15.0% N, 9.0% P, 12.0% K, 2.5% MgO and microelements; Osmocote Exact, ICL, Israel) per liter. Plants in the NF group were grown without the addition of any supplemented fertilizer. Each treatment group (F and NF) contained the same number of plants with the same paternal or maternal origin. Plants were further divided into two blocks containing the same number of male and female plants from each treatment group.

Whole plants were harvested four times per year (March, June, September, and December) in 2014 and 2015. Sampling dates were linked to plant phenology, with the first (March) occurring before strobili opening; the second (June) occurring immediately after pollination, during the most intensive period of vegetative growth; the third (September/October) occurring during biomass allocation primarily to roots; the fourth (December) occurring when plants were dormant. This pattern of sampling was repeated in both years. Plants did not exhibit presence of strobili in the first year of collection (2014) but all of the harvested plants exhibited cone initiation and strobili opening in the second year (2015). A total of 24 individuals were harvested on each sampling date. The same number of fertilized and nonfertilized, female and male plants were harvested each time. Three individuals from each parental origin were designated for collection at each sampling time (3 individuals × 2 sexes × 2 treatments × 2 blocks = 24).

Whole plants were harvested and dried at 65 °C for 72 h. All needles were separated from their respective shoots and used in downstream analyses. Needles were ground to a fine powder prior to use with the aid of a Mikro-Feinmühle Culatti mill (IKA Labortechnik, Staufen, Germany).

### 4.3. Microclimate

Four EL-USB-2+ data loggers (Lascar electronics, Wiltshire, United Kingdom) were placed near the top of plants to monitor temperature and humidity. Meteorological parameters were measured every hour over the entire two-year duration of the study. Monthly mean, minimal, and maximal temperatures were calculated, as well as monthly mean relative humidity values, for the entire two-year sampling period (Table 1). Monthly mean temperatures ranged between 18.14 ± 7.95 °C (mean ± SE) in June 2014 and −2.00 ± 5.97 °C in December 2015. The highest and lowest temperatures occurred in September and December 2015, respectively. The difference between the highest and lowest monthly temperature throughout the entire study was 60 °C. The lowest relative humidity occurred in June 2014 (RHmin= 69.70 ± 22.49) and the highest in December 2014 (RHmax = 92.21 ± 8.37). The difference between the lowest and the highest mean relative humidity throughout the study was 22.51%.

### 4.4. Sugars and Starch

A modification of the methods described by Haissig and Dickson [61], as well as Hansen and Møller [62], were used to determine the concentration of total nonstructural carbohydrates (TNC) and starch, as previously described in Oleksyn et al. [63]. Carbohydrates were extracted from powdered needle tissue in a 12:5:3 ratio solution of methanol:chloroform:water. The concentration of soluble sugars in the extracts was determined colorimetrically using anthrone reagent and measured at 625 nm, while starch concentration was determined in tissue residue by enzymatically converting the starch to glucose with amyloglucosidase. Absorbance was measured at 450 nm after a 30 min incubation at 25 °C in peroxidase-glucose oxidase-odianisidine dihydrochloride. The concentrations of soluble sugars and starch are expressed as a percentage of glucose per g of needle dry mass. Glucose standards were used to generate a standard curve using linear regression and the standard curve was used to estimate the concentration of soluble sugars in the samples.

### 4.5. Elemental Analysis

The concentration of a variety of elements was determined in powdered, leaf-tissue samples. The percentage of nitrogen and carbon in samples was determined using an Elemental Combustion System CHNS/O Analyser 2400 Series II (Perkin Elmer; Costech Analytical Technologies Inc., Valencia, CA, USA). The percentage of phosphorus, potassium, calcium, and magnesium in the samples was determined using an inductively coupled plasma–optical emission spectroscope (ICP–OES). Prior analysis samples were mineralized in nitric acid. Calibration was conducted by external standard method according to the standard PN-EN ISO 11885:2009 [64].

### 4.6. Total Phenols

A total of 0.1 g of tissue powder was used to determine the concentration of total phenols. Samples were boiled for 15 min in 95% ethanol and 10 min in 80% ethanol. Folin–Ciocalteu Phenol Reagent (Sigma F-9252) was used and the concentration of total phenols was determined spectrophotometrically by measuring absorbance at 660 nm as described by Johnson and Schaal [65] and modified by Singleton and Rossi [66]. The concentration of total phenolic compounds (TPhC) is expressed as µmol of chlorogenic acid per g^−1^ dry mass.

### 4.7. Statistical Analysis

Data were analyzed using JMP^®^ 15.1 Pro software (SAS Institute Inc., Cary, NC, USA, 1989–2019). Prior statistical analysis of results obtained as percentages were calculated with Bliss correction [67]. Outliers were identified using methods as described by Dean et al. [68] and studentized residual plots and residual normal quantile plots were analyzed. Homogeneity of variances was checked using Levene’s test and all data were tested for normality with the Shapiro–Wilk test. Logarythmization was applied to data that did not exhibit a normal distribution. Data originating from each measured term were analyzed using a two-way ANOVA since a different set of individuals were evaluated at each sampling term. Sex and soil fertilization treatment were used as fixed effects. The number of individual nested in a block and in the number of maternal plants were treated as random effects. A Tukey’s HSD test was used to compare mean values when variability occurred. The presented data represent the mean ± standard error (SE) and differences between means were determined to be statistically significant at *p* < 0.05.

## 5. Conclusions

In the present study, we confirmed that males and females have different strategies of resource allocation, and that females invest more resources in storage and defense relative to males. It also appeared that this allocation pattern probably represents a difference in allocation strategy rather than a difference in the response to stress, because different levels of soil fertilization (fertilization and nonfertilization) in most cases did not result in differences in sex-specific responses. Differences between the sexes were not evident over the entire course of the study, and were only evident at some sampling time points. Notably, in some cases, the higher vs. lower levels of measured compounds were completely reversed in male and female plants at different time points of the study. These data suggest that long-term studies are critical for clearly assessing differences in resource allocation and stress response in male and female plants of dioecious species. Higher concentrations of macroelements (except C) in leaves of males can indicate differences in allocation and probably reflects their deposition in developing seeds in female plants rather than in leaves. The highest values of TPhC occurred during colder months which suggests that these compounds are involved in abiotic stress response in *J. communis*.

Our results showed differences in sexual dimorphism within chemical composition of male and female individuals of *J. communis*. However, it might not be enough to formulate undeniable conclusions, as we focused only on the needles and results of the analysis, which takes into account also shoots and roots might be different to some extent. Moreover, results were obtained for rooted shoots, few years old, and different values might be obtained for mature trees, which have invested in reproduction for a longer period.

Nevertheless, this study not only helped to understand the biology of *J. communis* but also showed the significance of long-term analysis for proper assessing of secondary sexual dimorphism. Our results show that studies concerning ecophysiological differences between sexes should include the influence of different reproductive roles of males and females. Those roles are highly connected with seasons when specific events in reproduction and allocation of biomass occurs and it should be included in experimental design to correctly understand the biology of dioecious species.

## Figures and Tables

**Table 1:** Air temperature (mean monthly, minimum, and maximum monthly temperatures) and mean monthly relative humidity monitored near the top of *J. communis* plants throughout the two-year study. Data were registered hourly and are presented as a mean ± SE (*n* = 745).

Dates		Monthly Temperature (°C)			Relative Air Humidity (%)
		Mean	Minimum	Maximum	
I year	March 2014	7.47 ± 7.97	−6.0	29.5	76.51 ± 21.43
	June 2014	18.14 ± 7.95	4.0	40.5	69.70 ± 22.49
	September 2014	16.49 ± 7.32	−1.0	36.0	78.82 ± 20.82
	December 2014	1.58 ± 3.33	−8.0	14.5	92.21 ± 8.37
II year	March 2015	5.68 ± 6.53	−8.5	26.0	76.99 ± 20.54
	June 2015	17.89 ± 7.95	4.0	43.0	72.34 ± 23.32
	September 2015	15.10 ± 7.31	0.5	44.5	76.31 ± 19.13
	December 2015	−2.00 ± 5.97	−15.5	13.5	91.37 ± 8.85

## Supplementary Materials

Supplementary materials are available online at https://www.mdpi.com/1422-0067/21/21/8094/s1.

## Abbreviations

TPhC	Total phenolic compounds
TNC	Total nonstructural carbohydrates
