# Supplementary material for: Sexual Dimorphism in the Chemical Composition of Male and Female in the Dioecious Tree, Juniperus communis L., Growing under Different Nutritional Conditions
Source: Int J Mol Sci. 2020 Oct 30;21(21):8094. doi: 10.3390/ijms21218094 (PMC7663750; doi:10.3390/ijms21218094)
Supplement: Supplementary file 1 [file ijms-21-08094-s001.zip › ijms-961554-supplementary/Table S1.docx]

**Supplementary file**

**Authors:** Mariola Rabska, Emilia Pers-Kamczyc, Roma Żytkowiak, Dawid Adamczyk, Grzegorz Iszkuło

**Title of manuscript**: Sexual dimorphism in the chemical composition of male and female in the dioecious tree, *Juniperus communis* L., growing under different nutritional conditions

**Table S1.** Changes with time in analysed parameters in needles of *J.communis* male and female individuals growing with or without soil fertilization. Data are means with standard errors (SE, *n* = 24). Results for: soluble sugars (%), starch (%), total non-structural carbohydrates (TNC, %), total phenolic compounds (TPhC, µmol/g^-1^ dry mass), carbon (%), nitrogen (%), C:N ratio, phosphorus (%), potassium (%), calcium (%) and magnesium (%).

| Parameter | Year | Month | Non-fertilized | | | | Fertilized | | | |
| --- | --- | --- | --- | --- | --- | --- | --- | --- | --- | --- |
|  |  |  | Female | | Male | | Female | | Male | |
|  |  |  | Mean | SE | Mean | SE | Mean | SE | Mean | SE |
| soluble sugars  (%) | 2014 | III | 8.185 | 0.604 | 8.212 | 0.441 | 8.534 | 0.493 | 8.419 | 0.283 |
|  | 2014 | VI | 5.827 | 0.280 | 6.042 | 0.522 | 6.104 | 0.396 | 6.112 | 0.464 |
|  | 2014 | IX | 7.276 | 0.475 | 7.143 | 0.499 | 6.813 | 0.515 | 6.756 | 0.328 |
|  | 2014 | XII | 7.783 | 0.479 | 7.775 | 0.348 | 7.764 | 0.371 | 7.102 | 0.248 |
|  | 2015 | III | 7.024 | 0.353 | 7.938 | 1.886 | 7.136 | 0.685 | 7.445 | 2.077 |
|  | 2015 | VI | 7.050 | 0.230 | 5.911 | 0.268 | 7.169 | 0.513 | 6.425 | 0.372 |
|  | 2015 | IX | 6.969 | 0.439 | 6.609 | 0.460 | 6.038 | 0.363 | 6.717 | 0.333 |
|  | 2015 | XII | 7.451 | 0.292 | 7.824 | 0.349 | 6.938 | 0.452 | 7.840 | 0.223 |
| starch (%) | 2014 | III | 6.274 | 0.542 | 3.853 | 0.813 | 2.021 | 0.304 | 1.483 | 0.208 |
|  | 2014 | VI | 10.123 | 1.115 | 5.192 | 0.668 | 6.068 | 1.153 | 4.563 | 0.629 |
|  | 2014 | IX | 0.459 | 0.011 | 0.452 | 0.011 | 0.458 | 0.013 | 0.466 | 0.010 |
|  | 2014 | XII | 0.455 | 0.004 | 0.444 | 0.007 | 0.460 | 0.008 | 0.443 | 0.007 |
|  | 2015 | III | 4.992 | 0.765 | 5.715 | 0.887 | 2.633 | 0.605 | 2.592 | 0.531 |
|  | 2015 | VI | 4.440 | 1.594 | 1.676 | 0.600 | 3.386 | 0.743 | 1.132 | 0.373 |
|  | 2015 | IX | 0.429 | 0.006 | 0.417 | 0.008 | 0.430 | 0.002 | 0.425 | 0.006 |
|  | 2015 | XII | 0.417 | 0.009 | 0.421 | 0.009 | 0.430 | 0.007 | 0.417 | 0.005 |
| TNC (%) | 2014 | III | 12.551 | 1.801 | 12.745 | 0.881 | 10.554 | 0.322 | 9.902 | 0.217 |
|  | 2014 | VI | 15.950 | 1.208 | 11.233 | 1.064 | 11.442 | 1.166 | 10.080 | 0.875 |
|  | 2014 | IX | 7.780 | 0.497 | 8.417 | 1.037 | 7.695 | 0.825 | 7.222 | 0.327 |
|  | 2014 | XII | 8.663 | 0.580 | 8.219 | 0.348 | 8.224 | 0.368 | 7.544 | 0.254 |
|  | 2015 | III | 15.396 | 3.695 | 16.980 | 3.800 | 9.769 | 0.756 | 10.037 | 2.192 |
|  | 2015 | VI | 11.490 | 1.524 | 8.004 | 0.531 | 10.555 | 0.761 | 8.604 | 1.213 |
|  | 2015 | IX | 7.430 | 0.423 | 7.988 | 1.022 | 7.224 | 0.812 | 7.142 | 0.333 |
|  | 2015 | XII | 8.347 | 0.526 | 8.246 | 0.351 | 7.993 | 0.730 | 8.258 | 0.221 |
| TPhC  (µmol/  g^-1^ dry mass) | 2014 | III | 103.329 | 7.775 | 90.638 | 3.589 | 93.103 | 5.389 | 85.422 | 3.689 |
|  | 2014 | VI | 77.489 | 7.006 | 91.590 | 6.980 | 87.440 | 9.485 | 87.804 | 2.378 |
|  | 2014 | IX | 144.285 | 13.053 | 118.057 | 7.493 | 128.062 | 5.480 | 102.666 | 5.173 |
|  | 2014 | XII | 242.796 | 12.663 | 203.932 | 15.044 | 209.901 | 8.897 | 195.213 | 9.543 |
|  | 2015 | III | 103.892 | 5.144 | 97.577 | 6.707 | 86.774 | 9.628 | 72.848 | 4.279 |
|  | 2015 | VI | 95.081 | 7.442 | 87.122 | 4.551 | 83.002 | 2.812 | 78.615 | 4.893 |
|  | 2015 | IX | 97.472 | 4.448 | 90.656 | 4.515 | 96.336 | 4.572 | 76.847 | 3.246 |
|  | 2015 | XII | 190.902 | 9.192 | 206.807 | 5.551 | 187.576 | 4.955 | 166.467 | 7.875 |
| C (%) | 2014 | III | 47.466 | 0.207 | 47.318 | 0.146 | 47.950 | 0.162 | 47.925 | 0.083 |
|  | 2014 | VI | 47.515 | 0.228 | 46.943 | 0.133 | 47.426 | 0.243 | 47.577 | 0.313 |
|  | 2014 | IX | 48.794 | 0.126 | 48.127 | 0.166 | 49.154 | 0.204 | 48.593 | 0.190 |
|  | 2014 | XII | 49.200 | 0.158 | 48.548 | 0.148 | 49.137 | 0.119 | 48.942 | 0.175 |
|  | 2015 | III | 47.947 | 0.150 | 47.304 | 0.168 | 48.492 | 0.268 | 48.368 | 0.168 |
|  | 2015 | VI | 47.470 | 0.122 | 47.570 | 0.131 | 47.782 | 0.072 | 47.872 | 0.118 |
|  | 2015 | IX | 49.383 | 0.339 | 48.507 | 0.059 | 49.150 | 0.159 | 48.922 | 0.283 |
|  | 2015 | XII | 49.288 | 0.192 | 48.925 | 0.147 | 49.550 | 0.149 | 49.183 | 0.163 |
| N (%) | 2014 | III | 1.275 | 0.080 | 1.527 | 0.141 | 2.042 | 0.254 | 2.478 | 0.080 |
|  | 2014 | VI | 0.905 | 0.058 | 1.230 | 0.113 | 1.747 | 0.079 | 1.765 | 0.061 |
|  | 2014 | IX | 1.163 | 0.078 | 1.207 | 0.055 | 2.132 | 0.070 | 2.218 | 0.076 |
|  | 2014 | XII | 1.047 | 0.039 | 1.195 | 0.082 | 2.170 | 0.085 | 2.178 | 0.098 |
|  | 2015 | III | 1.195 | 0.092 | 1.040 | 0.042 | 2.343 | 0.068 | 2.137 | 0.191 |
|  | 2015 | VI | 1.107 | 0.110 | 1.107 | 0.057 | 1.925 | 0.068 | 1.927 | 0.094 |
|  | 2015 | IX | 1.398 | 0.065 | 1.415 | 0.073 | 2.143 | 0.047 | 2.425 | 0.096 |
|  | 2015 | XII | 1.218 | 0.037 | 1.282 | 0.079 | 2.182 | 0.102 | 2.037 | 0.113 |
| C:N | 2014 | III | 37.389 | 2.618 | 32.449 | 3.223 | 27.014 | 5.784 | 19.447 | 0.676 |
|  | 2014 | VI | 53.560 | 3.320 | 39.848 | 3.759 | 27.129 | 1.448 | 27.116 | 0.954 |
|  | 2014 | IX | 42.484 | 4.104 | 40.302 | 1.860 | 23.063 | 0.872 | 22.020 | 0.675 |
|  | 2014 | XII | 47.322 | 1.724 | 41.504 | 2.568 | 22.811 | 0.864 | 22.698 | 1.036 |
|  | 2015 | III | 41.525 | 3.691 | 44.616 | 1.606 | 20.776 | 0.575 | 24.804 | 3.802 |
|  | 2015 | VI | 44.965 | 5.249 | 42.732 | 2.452 | 24.983 | 0.924 | 25.137 | 1.196 |
|  | 2015 | IX | 36.370 | 1.865 | 34.779 | 1.955 | 23.117 | 0.617 | 20.335 | 0.824 |
|  | 2015 | XII | 40.651 | 1.307 | 38.906 | 2.432 | 22.893 | 1.106 | 24.552 | 1.471 |
| P (%) | 2014 | III | 0.159 | 0.016 | 0.211 | 0.019 | 0.215 | 0.007 | 0.263 | 0.016 |
|  | 2014 | VI | 0.120 | 0.003 | 0.153 | 0.014 | 0.155 | 0.013 | 0.174 | 0.010 |
|  | 2014 | IX | 0.176 | 0.009 | 0.198 | 0.013 | 0.182 | 0.007 | 0.211 | 0.013 |
|  | 2014 | XII | 0.159 | 0.006 | 0.197 | 0.010 | 0.216 | 0.014 | 0.274 | 0.013 |
|  | 2015 | III | 0.178 | 0.013 | 0.140 | 0.007 | 0.273 | 0.012 | 0.255 | 0.025 |
|  | 2015 | VI | 0.156 | 0.012 | 0.179 | 0.009 | 0.152 | 0.004 | 0.150 | 0.009 |
|  | 2015 | IX | 0.225 | 0.006 | 0.223 | 0.016 | 0.184 | 0.004 | 0.224 | 0.006 |
|  | 2015 | XII | 0.197 | 0.014 | 0.187 | 0.008 | 0.211 | 0.027 | 0.176 | 0.009 |
| K (%) | 2014 | III | 0.696 | 0.025 | 0.759 | 0.044 | 0.751 | 0.018 | 0.818 | 0.032 |
|  | 2014 | VI | 0.716 | 0.022 | 0.877 | 0.033 | 0.717 | 0.023 | 0.816 | 0.035 |
|  | 2014 | IX | 0.797 | 0.027 | 0.798 | 0.027 | 0.814 | 0.009 | 0.987 | 0.042 |
|  | 2014 | XII | 0.636 | 0.030 | 0.706 | 0.016 | 0.530 | 0.031 | 0.608 | 0.028 |
|  | 2015 | III | 0.636 | 0.040 | 0.652 | 0.020 | 0.744 | 0.031 | 0.826 | 0.029 |
|  | 2015 | VI | 0.718 | 0.024 | 0.796 | 0.033 | 0.677 | 0.031 | 0.679 | 0.011 |
|  | 2015 | IX | 0.648 | 0.027 | 0.704 | 0.022 | 0.593 | 0.033 | 0.680 | 0.033 |
|  | 2015 | XII | 0.524 | 0.021 | 0.552 | 0.023 | 0.558 | 0.036 | 0.565 | 0.034 |
| Ca (%) | 2014 | III | 1.249 | 0.060 | 0.982 | 0.048 | 0.865 | 0.027 | 0.761 | 0.017 |
|  | 2014 | VI | 0.825 | 0.051 | 1.091 | 0.037 | 0.709 | 0.061 | 0.646 | 0.023 |
|  | 2014 | IX | 1.263 | 0.165 | 1.231 | 0.115 | 0.677 | 0.062 | 0.783 | 0.039 |
|  | 2014 | XII | 1.194 | 0.127 | 1.522 | 0.099 | 0.804 | 0.073 | 0.887 | 0.031 |
|  | 2015 | III | 1.176 | 0.084 | 1.293 | 0.029 | 0.645 | 0.020 | 0.733 | 0.052 |
|  | 2015 | VI | 1.143 | 0.115 | 1.147 | 0.062 | 0.600 | 0.069 | 0.587 | 0.048 |
|  | 2015 | IX | 1.052 | 0.056 | 1.148 | 0.069 | 0.656 | 0.006 | 0.609 | 0.049 |
|  | 2015 | XII | 1.055 | 0.083 | 1.226 | 0.048 | 0.606 | 0.066 | 0.650 | 0.030 |
| Mg (%) | 2014 | III | 0.111 | 0.008 | 0.130 | 0.006 | 0.115 | 0.006 | 0.118 | 0.003 |
|  | 2014 | VI | 0.109 | 0.005 | 0.138 | 0.004 | 0.103 | 0.008 | 0.125 | 0.006 |
|  | 2014 | IX | 0.155 | 0.007 | 0.216 | 0.009 | 0.167 | 0.011 | 0.231 | 0.009 |
|  | 2014 | XII | 0.196 | 0.015 | 0.219 | 0.011 | 0.197 | 0.016 | 0.226 | 0.014 |
|  | 2015 | III | 0.184 | 0.010 | 0.177 | 0.010 | 0.190 | 0.004 | 0.220 | 0.015 |
|  | 2015 | VI | 0.187 | 0.023 | 0.173 | 0.004 | 0.178 | 0.021 | 0.185 | 0.014 |
|  | 2015 | IX | 0.215 | 0.034 | 0.215 | 0.010 | 0.233 | 0.026 | 0.238 | 0.014 |
|  | 2015 | XII | 0.207 | 0.017 | 0.196 | 0.012 | 0.204 | 0.025 | 0.242 | 0.015 |
