# Supplementary material for: Sexual Dimorphism in the Chemical Composition of Male and Female in the Dioecious Tree, Juniperus communis L., Growing under Different Nutritional Conditions
Source: Int J Mol Sci. 2020 Oct 30;21(21):8094. doi: 10.3390/ijms21218094 (PMC7663750; doi:10.3390/ijms21218094)
Supplement: Supplementary file 1 [file ijms-21-08094-s001.zip › ijms-961554-supplementary/Table S2.docx]

**Supplementary file**

**Authors:** Mariola Rabska, Emilia Pers-Kamczyc, Roma Żytkowiak, Dawid Adamczyk, Grzegorz Iszkuło

**Title of manuscript**: Sexual dimorphism in the chemical composition of male and female in the dioecious tree, *Juniperus communis* L., growing under different nutritional conditions

**Table S2.** ANOVA results for parameters analysed during two years in needles of *J.communis* male and female individuals growing with or without soil fertilization. Effects of sex, soil fertilization and interaction were shown and results for: soluble sugars (%), starch (%), total non-structural carbohydrates (TNC, %), total phenolic compounds (TPhC, µmol/g^-1^ dry mass), carbon (%), nitrogen (%), C:N ratio, phosphorus (%), potassium (%), calcium (%) and magnesium (%). P<0.05 was marked in red and bold.

|  |  |  | soluble sugars (%) | | starch (%) | | TNC (%) | | TPhC (µmol/g^-1^ dry mass) | | C (%) | | N (%) | | C:N ratio | | P (%) | | K (%) | | Ca (%) | | Mg (%) | |
| --- | --- | --- | --- | --- | --- | --- | --- | --- | --- | --- | --- | --- | --- | --- | --- | --- | --- | --- | --- | --- | --- | --- | --- | --- |
| year | month | effects | F ratio | P | F ratio | P | F ratio | P | F ratio | P | F ratio | P | F ratio | P | F ratio | P | F ratio | P | F ratio | P | F ratio | P | F ratio | P |
| 2014 |  |  |  |  |  |  |  |  |  |  |  |  |  |  |  |  |  |  |  |  |  |  |  |  |
|  | III | sex | 0.00 | 0.9557 | 9.19 | **0.0072** | 2.05 | 0.1689 | 4.17 | 0.0562 | 0.33 | 0.5753 | 4.14 | **0.0560** | 2.83 | 0.1089 | 10.67 | **0.0041** | 4.17 | 0.0545 | 23.23 | **0.0001** | 3.58 | 0.0749 |
|  |  | fertilization | 0.41 | 0.5311 | 50.66 | **<0.0001** | 21.00 | **0.0002** | 2.39 | 0.1393 | 12.97 | **0.0019** | 77.98 | **<0.0001** | 47.94 | **<0.0001** | 13.03 | **0.0019** | 3.44 | 0.0786 | 63.35 | **<0.0001** | 0.52 | 0.4822 |
|  |  | sex*fertilization | 0.02 | 0.8857 | 2.19 | 0.1561 | 0.18 | 0.6774 | 0.25 | 0.6218 | 0.16 | 0.6898 | 0.20 | 0.6575 | 0.66 | 0.4279 | 0.17 | 0.6865 | 0.00 | 0.9716 | 3.30 | 0.0861 | 1.57 | 0.2255 |
|  |  |  |  |  |  |  |  |  |  |  |  |  |  |  |  |  |  |  |  |  |  |  |  |  |
|  | IV | sex | 0.05 | 0.8315 | 11.61 | **0.0031** | 8.11 | **0.0107** | 1.07 | 0.3142 | 0.78 | 0.3897 | 7.41 | **0.0140** | 13.77 | **0.0016** | 6.56 | **0.0186** | 20.57 | **0.0002** | 3.77 | 0.0679 | 18.32 | **0.0004** |
|  |  | fertilization | 0.17 | 0.6887 | 6.14 | **0.0234** | 7.12 | **0.0156** | 0.19 | 0.6644 | 1.29 | 0.2694 | 105.87 | **<0.0001** | 68.37 | **<0.0001** | 7.37 | **0.0134** | 1.07 | 0.3129 | 34.80 | **<0.0001** | 2.65 | 0.1193 |
|  |  | sex*fertilization | 0.05 | 0.8342 | 2.90 | 0.1055 | 2.32 | 0.1455 | 0.96 | 0.3385 | 2.28 | 0.1476 | 6.05 | **0.0242** | 12.02 | **0.0027** | 0.53 | 0.4733 | 1.08 | 0.3122 | 10.64 | **0.0043** | 0.27 | 0.6097 |
|  |  |  |  |  |  |  |  |  |  |  |  |  |  |  |  |  |  |  |  |  |  |  |  |  |
|  | IX | sex | 0.03 | 0.8594 | 0.00 | 0.9882 | 0.00 | 0.9751 | 10.49 | **0.0046** | 12.11 | **0.0027** | 0.14 | 0.7125 | 0.03 | 0.8587 | 5.99 | **0.0255** | 8.87 | **0.0080** | 0.31 | 0.5849 | 49.08 | **<0.0001** |
|  |  | fertilization | 0.83 | 0.3738 | 0.34 | 0.5677 | 1.12 | 0.3047 | 3.94 | 0.0628 | 5.49 | **0.0309** | 227.75 | **<0.0001** | 186.14 | **<0.0001** | 0.84 | 0.3735 | 12.99 | **0.0020** | 26.43 | **<0.0001** | 2.21 | 0.1530 |
|  |  | sex*fertilization | 0.01 | 0.9084 | 0.48 | 0.4985 | 0.29 | 0.5990 | 0.00 | 0.9589 | 0.09 | 0.7656 | 0.68 | 0.4213 | 0.35 | 0.5608 | 0.09 | 0.7692 | 8.54 | **0.0091** | 0.55 | 0.4683 | 0.01 | 0.9388 |
|  |  |  |  |  |  |  |  |  |  |  |  |  |  |  |  |  |  |  |  |  |  |  |  |  |
|  | XII | sex | 0.85 | 0.3678 | 4.50 | **0.0465** | 0.92 | 0.3499 | 5.15 | **0.0350** | 7.81 | **0.0112** | 0.52 | 0.4783 | 1.90 | 0.1845 | 19.89 | **0.0003** | 7.50 | **0.0126** | 6.11 | **0.0231** | 3.45 | 0.0782 |
|  |  | fertilization | 0.91 | 0.3512 | 0.11 | 0.7459 | 0.89 | 0.3564 | 3.11 | 0.0938 | 1.19 | 0.2889 | 253.94 | **<0.0001** | 256.17 | **<0.0001** | 39.28 | **<0.0001** | 14.23 | **0.0012** | 38.71 | **<0.0001** | 0.07 | 0.7943 |
|  |  | sex*fertilization | 0.85 | 0.3684 | 0.19 | 0.6710 | 0.87 | 0.3632 | 1.05 | 0.3183 | 2.27 | 0.1473 | 0.40 | 0.5331 | 1.68 | 0.2101 | 0.41 | 0.5317 | 0.07 | 0.7937 | 1.48 | 0.2384 | 0.04 | 0.8346 |
| 2015 |  |  |  |  |  |  |  |  |  |  |  |  |  |  |  |  |  |  |  |  |  |  |  |  |
|  | III | sex | 0.01 | 0.9270 | 0.15 | 0.7006 | 0.04 | 0.8403 | 2.10 | 0.1634 | 3.64 | 0.0725 | 1.74 | 0.2023 | 7.20 | **0.0152** | 4.45 | **0.0483** | 2.58 | 0.1254 | 4.58 | **0.0455** | 1.18 | 0.2908 |
|  |  | fertilization | 0.06 | 0.8088 | 14.37 | **0.0011** | 8.44 | **0.0099** | 8.98 | **0.0074** | 16.05 | **0.0008** | 272.79 | **<0.0001** | 200.31 | **<0.0001** | 52.41 | **<0.0001** | 21.48 | **0.0002** | 128.43 | **<0.0001** | 5.93 | **0.0255** |
|  |  | sex*fertilization | 0.08 | 0.7796 | 0.17 | 0.6883 | 1.75 | 0.2028 | 0.30 | 0.5920 | 1.67 | 0.2124 | 1.25 | 0.2780 | 6.43 | **0.0207** | 0.70 | 0.4136 | 0.94 | 0.3453 | 0.01 | 0.9361 | 3.06 | 0.0975 |
|  |  |  |  |  |  |  |  |  |  |  |  |  |  |  |  |  |  |  |  |  |  |  |  |  |
|  | IV | sex | 6.49 | **0.0197** | 8.10 | **0.0103** | 11.28 | **0.0033** | 1.41 | 0.2487 | 0.73 | 0.4046 | 0.00 | 0.9530 | 2.51 | 0.1317 | 1.10 | 0.3075 | 2.13 | 0.1606 | 0.00 | 0.9903 | 0.01 | 0.9233 |
|  |  | fertilization | 0.67 | 0.4232 | 0.42 | 0.5264 | 0.44 | 0.5150 | 3.93 | 0.0615 | 7.59 | **0.0130** | 89.34 | **<0.0001** | 163.97 | **<0.0001** | 2.71 | 0.1164 | 8.24 | **0.0098** | 54.65 | **<0.0001** | 0.01 | 0.9323 |
|  |  | sex*fertilization | 0.35 | 0.5604 | 0.01 | 0.9422 | 0.01 | 0.9063 | 0.12 | 0.7346 | 0.00 | 0.9645 | 0.00 | 0.9460 | 2.81 | 0.1118 | 1.88 | 0.1867 | 1.73 | 0.2036 | 0.02 | 0.8962 | 0.36 | 0.5553 |
|  |  |  |  |  |  |  |  |  |  |  |  |  |  |  |  |  |  |  |  |  |  |  |  |  |
|  | IX | sex | 0.18 | 0.6780 | 1.94 | 0.1812 | 0.14 | 0.7099 | 9.58 | **0.0066** | 6.03 | **0.0251** | 3.32 | 0.0835 | 6.08 | **0.0234** | 3.81 | 0.0659 | 5.99 | **0.0238** | 0.05 | 0.8219 | 0.10 | 0.7592 |
|  |  | fertilization | 1.02 | 0.3265 | 0.65 | 0.4322 | 1.15 | 0.2975 | 3.09 | 0.0967 | 0.16 | 0.6898 | 143.47 | **<0.0001** | 130.03 | **<0.0001** | 4.55 | **0.0462** | 1.93 | 0.1800 | 82.31 | **<0.0001** | 0.82 | 0.3755 |
|  |  | sex*fertilization | 1.72 | 0.2058 | 0.39 | 0.5430 | 1.89 | 0.1860 | 2.22 | 0.1543 | 2.07 | 0.1680 | 2.49 | 0.1301 | 0.00 | 0.9751 | 5.29 | **0.0330** | 0.29 | 0.5944 | 1.96 | 0.1778 | 0.00 | 0.9726 |
|  |  |  |  |  |  |  |  |  |  |  |  |  |  |  |  |  |  |  |  |  |  |  |  |  |
|  | XII | sex | 3.73 | 0.0694 | 0.33 | 0.5730 | 3.60 | 0.0741 | 0.14 | 0.7172 | 4.82 | **0.0407** | 0.14 | 0.7132 | 0.10 | 0.7525 | 1.69 | 0.2078 | 0.38 | 0.5432 | 3.02 | 0.0983 | 0.67 | 0.4217 |
|  |  | fertilization | 0.59 | 0.4517 | 0.34 | 0.5654 | 0.60 | 0.4469 | 9.52 | **0.0061** | 2.45 | 0.1342 | 103.05 | **<0.0001** | 141.18 | **<0.0001** | 0.00 | 0.9963 | 0.62 | 0.4392 | 68.47 | **<0.0001** | 1.43 | 0.2466 |
|  |  | sex*fertilization | 0.72 | 0.4079 | 1.22 | 0.2833 | 0.71 | 0.4105 | 6.84 | **0.0170** | 0.00 | 0.9921 | 1.37 | 0.2554 | 2.49 | 0.1321 | 0.47 | 0.5014 | 0.14 | 0.7160 | 0.59 | 0.4526 | 2.12 | 0.1620 |
